# Endoscopic vacuum therapy significantly improves clinical outcomes of anastomotic leakages after 2-stage, 3-stage, and transhiatal esophagectomies

**DOI:** 10.1007/s00423-023-02826-3

**Published:** 2023-02-15

**Authors:** Jonas Maier, A. Kandulski, N. E. Donlon, J. M. Werner, A. Mehrl, M. Müller, A. Doenecke, H. J. Schlitt, M. Hornung, A. R. R. Weiss

**Affiliations:** 1 Department of Surgery, University Hospital Regensburg, Franz-Josef-Strauss-Allee 11, 93053 Regensburg, Germany; 2 Department of Internal Medicine I, Gastroenterology, Hepatology, Endocrinology, Rheumatology, and Infectious Diseases, University Hospital Regensburg, Regensburg, Germany; 3 Department of Surgery, Mater Misericordiae University Hospital, Dublin 7, Ireland

**Keywords:** Endoscopic vacuum therapy, Anastomotic leak, Esophagectomy, Gastric tube reconstruction, Endoscopic vacuum-assisted closure therapy, Negative pressure wound therapy

## Abstract

**Background:**

Anastomotic leakages after esophagectomies continue to constitute significant morbidity and mortality. Intrathoracic anastomoses pose a high risk for mediastinitis, sepsis, and death, if a leak is not addressed timely and appropriately. However, there are no standardized treatment recommendations or algorithms as for how to treat these leakages.

**Methods:**

The study included all patients at the University Hospital Regensburg, who developed an anastomotic leakage after esophagectomy with gastric pull-up reconstruction from 2007 to 2022. Patients receiving conventional treatment options for an anastomotic leakage (stents, drainage tubes, clips, etc.) were compared to patients receiving endoscopic vacuum-assisted closure (eVAC) therapy as their mainstay of treatment. Treatment failure was defined as cervical esophagostomy formation or death.

**Results:**

In total, 37 patients developed an anastomotic leakage after esophagectomy with a gastric pull-up reconstruction. Twenty patients were included into the non-eVAC cohort, whereas 17 patients were treated with eVAC. Treatment failure was observed in 50% of patients (*n* = 10) in the non-eVAC cohort and in 6% of patients (*n* = 1) in the eVAC cohort (*p* < 0.05). The 90-day mortality in the non-eVAC cohort was 15% (*n* = 3) compared to 6% (*n* = 1) in the eVAC cohort. Cervical esophagostomy formation was required in 40% of cases (*n* = 8) in the non-eVAC cohort, whereas no patient in the eVAC cohort underwent cervical esophagostomy formation.

**Conclusion:**

eVAC therapy for leaking esophagogastric anastomoses appears to be superior to other treatment strategies as it significantly reduces morbidity and mortality. Therefore, we suggest eVAC as an essential component in the treatment algorithm for anastomotic leakages following esophagectomies, especially in patients with intrathoracic anastomoses.

## Introduction 

Anastomotic leakages continue to be a highly challenging complication in esophageal surgery. According to the literature, the risk of anastomotic leakage after esophagectomy ranges between 4 and 35% [1, 2]. The location of the anastomotic leakage is a significant factor in determining patient outcomes. Notwithstanding, cervical anastomoses bear a higher risk for leakage; the consequences of an intrathoracic (mediastinal) leakage are usually more devastating [3]. A leakage into the thoracic cavity typically leads to mediastinitis and severe pneumonia and contributes to the significant mortality rates in esophageal surgery. In contrast, cervical anastomotic leakages tend to frequently present as wound infections often only requiring external drainage [4, 5].

The clinical outcomes strongly depend on an early diagnosis and appropriate treatment, which can extent over several weeks or even months [4]. In the past, the mainstay of treatment was based on surgical repair, external drainage of sepsis via chest tubes, and interventional treatment modalities like endoscopic stent deployment or clipping. In 2008, endoscopic vacuum-assisted closure (eVAC) therapy was successfully applied in patients with anastomotic leakages after esophagectomies [6].

As in other vacuum-assisted wound therapies, eVAC cleans the defect by reducing the amount of exudative fluids and necrotic tissue, thus accelerating the healing process by contributing to a better local perfusion as well as through the formation of granulation tissue [7, 8]. Since then, eVAC therapy has grown in popularity in clinical practice. However, there are still no clear recommendations as to whether eVAC therapy should be preferred over other treatment modalities. At the University Hospital Regensburg, eVAC therapy was introduced in 2014/2015 and has been the mainstay of treatment in all patients that developed an anastomotic leakage after esophageal resections with gastric pull-up reconstruction since 2017. The genesis of this study was to compare the clinical outcomes of “conventional” treatment modalities with the results of eVAC therapy in the treatment of leaking esophagogastric anastomoses after gastric pull-up reconstruction.

## Methods

### Study population and design

All patients between 2007 and 2022 undergoing esophageal resections and reconstruction with a gastric conduit that developed an anastomotic leakage in the postoperative course were included in this study. An anastomotic leakage had to be verified by (CT-) contrast swallow, upper endoscopy (EGD), or by an intraoperatively observed dehiscence of the esophagogastric anastomosis. The data were collected retrospectively from medical files including ICU reports, OP notes, and discharge letters as well as from radiographic and endoscopic reports. The information was gathered from the University Hospital Regensburg’s institutional archive and database (SAP Version 7.50).

Demographical data such as age, gender, and body weight as well as the medical history were recorded. Data regarding neoadjuvant treatment, surgery, and complications in the postoperative course including the management of the anastomotic leakage were obtained for statistical evaluation. Patients were included in the eVAC cohort, if at least one complete cycle of eVAC therapy (3–4 days) was performed. Other treatment modalities such as placement of fully covered self-expanding metal stents (fcSEMS), external drainage tubes, or initial watch-and-wait therapy could be part of the overall treatment concept in the eVAC cohort, given, that eVAC therapy was applied for at least one full cycle. A surgical revision for drainage and lavage of the thoracic cavity was defined as complementary treatment in the eVAC and non-eVAC group, given, that an empyema or intrathoracic abscess/fluid collection was not amenable for interventional/endoscopic drainage. eVAC was performed using the Eso-SPONGE device (B. Braun SE; Melsungen, Germany) placed endoscopically in the thoracic cavity or intraluminal. A negative pressure gradient of − 125 mmHg was applied on the eVAC using V.A.C. ULTA system (3 M Deutschland GmbH). fcSEMS from TaeWoong Medical (Niti-S Esophageal Stent, Taewoong Medical, Gyeonggi-do, South Korea) and M.I. Tech, (Hanarostent, M.I. Tech, Seoul, South Korea) were used (diameter 20–22 mm, length 110–140 mm). The decision to use additional treatment modalities in the eVAC group, e.g., fcSEMS, was an individual decision in each case, based on the clinical appearance of the anastomotic leakage as well as on its development over the course of time and the experience of the treating surgeon/endoscopist.

A failure of treatment, defined by either formation of a cervical esophagostomy or death (30- and 90-day mortality) was the primary outcome parameter. Furthermore, as secondary outcomes, the cumulative hospital- and ICU-stay were recorded.

### Statistical analysis

For the statistical analysis, the IBM SPSS Statistics Version 29 for Mac OS was used. Continuous variables were presented as median values. The Mann–Whitney *U* test was performed for testing the statistical significance of parameters between the two groups. Relationships between categorical variables were evaluated using the chi-square test (two-sided) if 80% of the expected values were < = 5 and all expected values were > = 1. Alternatively, the Fisher’s exact test (two-sided) was used. Statistical significance was set as *p* < 0.05 for the research overall. Missing data were excluded from the calculations.

### Ethical approval

This study was approved by the ethics committee of the University of Regensburg (No. 21–2336-104). Individual consent of the patients involved was not required.

## Results

From 2007 to 2022 a total of 171 patients underwent a 2-stage, 3-stage, or transhiatal esophagectomy. Of these, 37 (22%) developed a radiologically, endoscopically, or intraoperatively proven anastomotic leakage. The demographical data and past medical history as well as the ASA score prior to esophageal surgery are listed in Table 1. Besides a higher incidence of coronary heart disease in eVAC group, no statistically significant differences were found between the two groups regarding the previous medical history.

**Table 1:** Demographics and medical history

Demographics and medical history	eVAC cohort (*n* = 17)	Non-eVAC cohort (*n* = 20)	*p*-value
Gender			n. s
Male	15 (88%)	17 (85%)	
Median age in years	61 [53.5; 69.5]	56 [52.3; 62]	n. s
Median BMI	25.4 [23.2; 30.9]	26.4 [24; 29.7]	n. s
Median ASA score	3	3	n. s
Previous abdominal surgery	6 (35%)	7 (35%)	n. s
Coronary heart disease	6 (35%)	0 (0%)	*p* < 0.05
Arterial hypertension	10 (59%)	9 (45%)	n. s
Peripheral arterial disease	1 (6%)	2 (10%)	n. s
Autoimmune diseases	2 (12%)	0	n. s
Sarcoidosis	1 (6%)		
Ulcerative colitis	1 (6%)		
GERD	2 (12%)	5 (25%)	n. s
Barrett esophagus	2 (12%)	6 (30%)	n. s
COPD	0	1 (5%)	n. s
Respiratory insufficiency	0	1 (5%)	n. s
Diabetes mellitus	5 (29%)	3 (15%)	n. s
Renal insufficiency	2 (12%)	2 (10%)	n. s
Hepatic disorder	4 (24%)	5 (25%)	n. s
Nicotine abuse	10 (63%)*	12 (60%)	n. s
Alcohol abuse	8 (50%)*	7 (35%)	n. s

^*^Information was not available for one patient (*n* = 16/17). Values in square brackets indicate interquartile ranges

### Pre- and postsurgical medication

No statistically significant difference in acetylsalicylic acid (ASA) use prior to esophagectomy was seen between the two cohorts. Gastric acid inhibitors (PPI) were used by 44% (*n* = 7) of patients in the eVAC- and by 78% (*n* = 14) of patients in the non-eVAC cohort (*p* < 0.05). After surgery, there was no statistically significant difference in anticoagulation or PPI use. ASA was applied in 41% (*n* = 7) of patients in the eVAC- and in 5% (*n* = 1) of patients in the non-eVAC cohort (*p* < 0.05). The post-surgical anticoagulation was evaluated according to a prophylactic or therapeutic treatment approach. There was no statistically significant difference between the two cohorts. The pre- and postsurgical medication is listed in Table 2.

**Table 2:** Pre- and postsurgical medication

Medication before esophagectomy	eVAC cohort (*n* = 16/17)	Non-eVAC cohort (*n* = 18/20)	*p*-value
PPI	7 (44%)	14 (78%)	*p* < 0.05
ASA	5 (31%)	2 (11%)	n. s
Medication after esophagectomy	(*n* = 17)	(*n* = 19/20)	
PPI	17 (100%)	16 (84%)	n. s
ASA	7 (41%)	1 (5%)	*p* < 0.05
Anticoagulation	17 (100%)	19 (100%)	n. s
Therapeutic	3 (18%)	7 (37%)	n. s
Prophylactic	14 (82%)	12 (63%)	n. s

For one patient in the eVAC and for two patients in the non-eVAC cohort, the presurgical medication was missing. For one patient in the non-eVAC cohort, the postsurgical medication was missing

### Neoadjuvant therapy

Neoadjuvant therapy had been applied in 94% of patients (*n* = 16) in the eVAC- and in 72% of patients (*n* = 13) in the non-eVAC cohort. One patient (6%) in the eVAC- and five patients (28%) in the non-eVAC cohort with an underlying malignancy had not received neoadjuvant treatment. Two patients (10%) in the non-eVAC cohort had undergone surgery for benign disease and were excluded from the calculations. Six patients (38%) in the eVAC cohort had been treated with chemotherapy only; in the non-eVAC cohort, chemotherapy had been applied in 8% of the patients (*n* = 1). Chemoradiotherapy (CRT) had been applied in 63% of patients (*n* = 10) in the eVAC- and in 92% of patients (*n* = 12) in non-eVAC cohort. The neoadjuvant treatment regimens are shown in Table 3.

**Table 3:** Neoadjuvant therapy

Neoadjuvant therapy	eVAC cohort (*n* = 17)	Non-eVAC cohort (*n* = 18/20*)	*p*-value
Patients receiving neoadjuvant therapy	16 (94%)	13 (72%)	n. s
Chemotherapy	6 (38%)	1 (8%)	n. s
FLOT	5 (83%)	0	
EOX	1 (17%)	0	
ECF	0	1 (100%)	
Chemoradiotherapy	10 (63%)	12 (92%)	n. s
5FU/cisplatin	3 (33%)	9 (75%)	
Carboplatin/paclitaxel	6 (67%)	2 (17%)	
5FU/oxaliplatin	0	1 (8%)	
Unknown	1	0	

^*^Two patients with benign diseases were excluded from the calculations

### Esophageal surgery

The main indication for esophagectomy among all patients was an esophageal or esophagogastric carcinoma (95%, *n* = 35). Ten percent (*n* = 2) of patients in the non-eVAC cohort had suffered from a benign condition (leiomyoma and Boerhaave syndrome). The respective histology is given in Table 4. Esophagectomies in the non-eVAC cohort were conducted from 2007 to 2017, in the eVAC cohort from 2014 to 2022. A hybrid surgical approach was most often used in the eVAC cohort (71%, *n* = 12). Patients in the non-eVAC group were commonly operated via an open surgical approach in 95% (*n* = 19; *p* < 0.05). The median duration of surgery was 6.7 h in the eVAC- and 6.3 h in the non-eVAC cohort. An intrathoracic anastomosis was performed in 53% of the patients (*n* = 9) in the eVAC cohort as compared to 95% of patients (*n* = 19) in the non-eVAC cohort. Consequently, 47% of patients (*n* = 8) in the eVAC cohort had received a cervical anastomosis in contrast to one patient (5%) in the non-eVAC cohort (*p* < 0.05). There were three fatalities (30- and 90-day mortality) in the non-eVAC cohort vs one in the eVAC cohort. The median duration of external drainage via chest tube(s) was 28 days in the eVAC- and 36 days in the non-eVAC cohort (*p* < 0.05). A nasogastric tube was placed for 40 days (median) in the eVAC- and for 13.5 days (median) in the non-eVAC cohort (*p* < 0.05).

**Table 4:** Esophageal surgery

Esophageal surgery	eVAC cohort (*n* = 17)	Non-eVAC cohort (*n* = 20)	*p*-value
Histology			
Adenocarcinoma	9 (53%)	9 (45%)	n. s
Squamous cell cancer	8 (47%)	9 (45%)	n. s
Benign etiology	0	2 (10%)	n. s
Years of esophageal surgery	2014–2022	2007–2017	
Surgical approach			*p* < 0.05
Open	5 (29%)	19 (95%)	
2-stage	4	18	
3-stage	0	1	
Transhiatal	1	0	
Hybrid	12 (71%)	0	
2-stage	5	0	
3-stage	5	0	
Transhiatal	2	0	
Totally minimal invasive	0	1 (5%)	
2-stage	0	1	
3-stage	0	0	
Transhiatal	0	0	
Median duration of surgery in minutes (hours)	399 [350; 453.5] (6.7)	378 [332; 419.3] (6.3)	n. s
Location of anastomosis			
Cervical	8 (47%)	1 (5%)	*p* < 0.05
Intrathoracic	9 (53%)	19 (95%)	*p* < 0.05

Values in square brackets indicate interquartile ranges

### Anastomotic leakage

The median time to diagnosis of the anastomotic leakage in the eVAC and non-eVAC cohort was 8 and 9.5 days, respectively. In 41% of patients in the eVAC cohort, rising inflammatory markers, specifically C-reactive protein (CRP) and white blood cell (WBC) count, were the reason for further examinations to confirm the suspected anastomotic leakage—either by a CT scan ± contrast swallow or a by primary endoscopic evaluation. The clinical deterioration of the patients and/or suspect secretions out of the chest tubes were the second most common cause for further diagnostics (35% of cases). In 18% of cases, the leakage was diagnosed through an EGD for other reasons (e.g., prophylactic intention, by chance). An elevation of the inflammatory markers triggered further examinations in 50% of cases in the non-eVAC cohort. Clinical deterioration was responsible in 45% and suspicious secretions out of the chest tubes in 25% of the cases. Evaluating the systemic inflammatory response of both cohorts from 5 days prior until the day of diagnosis of the anastomotic leakage, it could be seen that the WBC count was increasing by 34% as well as the CRP levels, which increased by 44% over the same time interval.

**Fig. 1:**
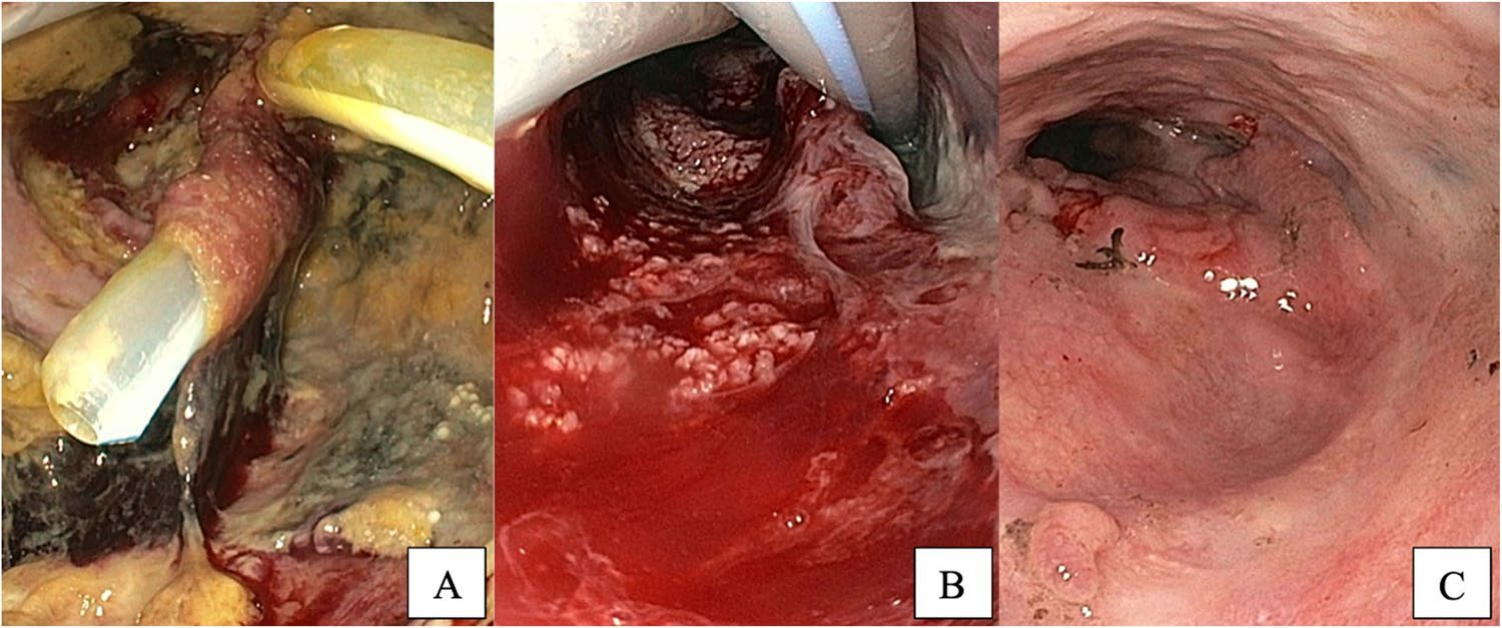
eVAC treatment of an anastomotic leakage after 2-stage esophagectomy with gastric pull-up reconstruction at day 0 (**A**), 21 (**B**), and 35 (**C**) after diagnosis

Fourteen patients in the Endo-Vac group received eVAC therapy in a therapeutic intention (82%). Three patients (18%) were treated with eVAC prophylactically, as the risk for leakage was considered to be high by the respective surgeon (due to a suspected, compromised blood supply of the distal part of the gastric conduit). In these cases, the eVAC was placed immediately after surgery. These three patients developed an endoscopically visible leak in the further course. Median duration of eVAC therapy was 18 days. The endosponge was replaced five times on average. In 77% of patients (*n* = 13), the endosponge was placed in an intracavitary position. An endoluminal application was performed in four patients (24%). The indicators for successful termination of the eVAC therapy can be seen in Table 5. No eVAC therapy had to be stopped because of deterioration of the anastomotic leakage. Figure 1 demonstrates the improvement of an anastomotic leakage after gastric pull-up reconstruction using eVAC therapy.

**Table 5:** Treatment of anastomotic leakage

Treatment of anastomotic leakage	eVAC cohort (*n* = 17)	Non-eVAC cohort (*n* = 20)	*p*-value
Days until diagnosis of AL	8 [6; 12.5]	9.5 [6.3; 12.8]	n. s
eVAC application	17 (100%)	−	−
Treatment approach			
Therapeutic approach	14 (82%)		−
Prophylactic approach	3 (18%)	−	
Endosponge placement			
Endoluminal	4 (24%)	−	−
Intracavitary	13 (77%)		
Median number of cycles	5 [2.5; 12]	−	−
Median duration of eVAC therapy in days	18 [12.5; 49]	−	−
Indications for termination of eVAC therapy (in %)	*n* = 17	−	−
Sufficient granulations	71%		
Size reduction of AL	59%		
Cleanliness of the defect	82%		
Visualization of fresh blood	24%		
Stent application	6 (35%)	17 (85%)	*p* < 0.05
Median duration of fcSEMS application in days	15 [10.8; 47.3]	35 [19.5; 53.5]	n. s
Surgical revision	5 (29%)	13 (65%)	*p* < 0.05
Duration of drainage via chest tube(s)	28 [7; 40.5]	36 [21.5; 47]	*p* < 0.05
Subsequent anastomotic stricture development	7 (41%)	3 (15%)	n. s

Values in square brackets indicate interquartile ranges

The application of fcSEMS was significantly less in the eVAC cohort (35%, *n* = 6) in comparison to the patients in the non-eVAC cohort (85%, *n* = 17; *p* < 0.05). The median duration of fcSEMS application was 15 days in the eVAC cohort and 35 days in the non-eVAC cohort. The main reason for stent application in the eVAC cohort was a significant residual, but by then clean cavity abutting the site of the anastomotic leakage (*n* = 3). It is of note that the stent was placed secondary to eVAC therapy in these cases. In one out of these three patients, one cycle of eVAC was performed prior to stent application. In the remaining two cases, four cycles of eVAC were performed in each case before stent deployment. A suspected but not confirmed anastomotic leakage was the rationale to apply a fcSEMS in the first place (*n* = 1). In this case, no eVAC therapy was performed beforehand. After stent extraction, the anastomotic leakage became obvious and an eVAC was placed inside the cavity. In one patient, a bronchial fistula was an indication for fcSEMS placement following two cycles of eVAC therapy, an anastomotic stricture due to local tumor recurrence in another patient. An anastomotic stricture following eVAC or conventional therapy was found in 41% (*n* = 7) of patients in the eVAC- and in 15% (*n* = 3) of the patients in the non-eVAC cohort (*p* = 0.136). A comparison of eVAC and non-eVAC treatment is shown in Table 5. Revision surgery was performed in five patients (29%) in the eVAC- and in thirteen patients (65%) in the non-eVAC cohort (*p* < 0.05). In the eVAC cohort, all five patients received surgical revision for drainage/lavage. In the non-eVAC cohort, ten patients (50%) received surgical drainage/lavage. An overstitch of the defect was performed in one patient in the eVAC- and in four patients in the non-eVAC cohort. A covering tissue flap was used in two patients in the non-eVAC cohort only. No patient in the eVAC cohort underwent cervical esophagostomy formation in contrast to eight patients (40%) in the non-eVAC group (*p* < 0.05). The indications for revision surgery in the eVAC group were intrathoracic abscess formation, pleural empyema, or other intrathoracic fluid collections not amenable for interventional/endoscopic drainage. Out of the five patients in the eVAC group undergoing revision surgery, one patient was planned for revision surgery on the same day of eVAC application; another patient underwent surgical revision before eVAC therapy was started. Two patients had the surgical revision during the first cycle of eVAC therapy (one day after eVAC application), another patient after nine cycles of eVAC.

### Hospitalization

The duration of hospital stay in the eVAC cohort was 65 days vs 64 days in the non-eVAC cohort. The cumulative ICU stay in the eVAC and non-eVAC cohort was 25 and 34.5 days, respectively. The cumulative ventilation time was 6.8 days in the eVAC- and 7.3 days in the non-eVAC cohort. The type of outpatient care after discharge from hospital can be seen in Table 6.

**Table 6:** Hospitalization

Hospitalization	eVAC cohort (*n* = 17)	Non-eVAC cohort (*n* = 20)	*p*-value
Cumulative hospital stay (incl. ICU) in days (median)	65 [52.5; 76.5]	64 [50.5; 97]	n. s
Cumulative ICU stay in days (median)	25 [13; 36]	34.5 [9.3; 56.3]	n. s
Cumulative duration of mechanical ventilation in days (median)	6.8 [1.7; 12.9]	7.3 [3.8; 13.3]	n. s
Care after discharge from hospital	(*n* = 17)	(*n* = 19/20)	n. s
Rehabilitation	2 (12%)	6 (32%)	
Home or ambulant care	14 (82%)	10 (53%)	
Other*	1 (6%)	3 (16%)	
Missing information	0	1	

^*^One patient in the eVAC cohort and three patients in the non-eVAC cohort died within 90 days post-surgery. Values in square brackets indicate interquartile ranges

### Clinical outcome

Negative clinical outcome, defined as death within the first 90 days after surgery or cervical esophagostomy formation, was significantly different. Fifty percent (*n* = 10) of patients in the non-eVAC cohort had a negative clinical outcome following anastomotic leakage (*p* < 0.05). Three patients (15%) died within the first 90 days after surgery, one of which had a cervical esophagostomy formation beforehand. All three patients died as a direct consequence of an insufficiently controlled intrathoracic anastomotic leakage: one patient died of multiorgan-failure caused by the anastomotic leakage. Another patient died of multiorgan-failure caused by the anastomotic leakage in combination with a middle cerebral artery infarction. The third patient died from hemorrhagic shock following a massive esophageal arterial bleeding at the leakage site, likely due to an aorto-esophageal fistula.

One patient in the eVAC cohort (6%) died of pneumonia 74 days after surgery in an external hospital. The cervical anastomotic leakage the patient was suffering from had healed completely 1 month beforehand and was not directly related to the patient’s death. Eight patients (40%) in the non-eVAC group required revision surgery with cervical esophagostomy formation, whereas no patient in the eVAC cohort had to undergo diversion surgery (*p* < 0.05). The subgroup analysis of patients with an intrathoracic anastomosis (*n* = 28) revealed a significantly higher rate of negative clinical outcomes in the non-eVAC cohort (47% vs 0%, *p* < 0.05). The negative clinical outcomes are shown in detail in Table 7.

**Table 7:** Negative clinical outcomes

Clinical outcome	eVAC cohort (*n* = 17)	Non-eVAC cohort (*n* = 20)	*p*-value
Negative clinical outcome	1 (6%)	10 (50%)	*p* < 0.05
30-day mortality	0	1 (5%)	n. s
90-day mortality	1 (6%)	3 (15%)	n. s
Cervical esophagostomy formation	0	8 (40%)	*p* < 0.05
Negative clinical outcome (subgroup with intrathoracic anastomosis)	0	9 (47.4%)	*p* < 0.05
30-day mortality	0	1 (5.3%)	n. s
90-day mortality	0	3 (15.8%)	n. s
Cervical esophagostomy formation	0	7 (36.8%)	*p* = 0.062

## Discussion

This study is one of the largest single-center studies to date comparing eVAC and non-eVAC treatment concepts for anastomotic leakages after 2-stage, 3-stage, and transhiatal esophagectomies. eVAC treatment was introduced at our institution in 2014/2015 and, after a transition period of three years, used in all cases of anastomotic leakages following esophageal resections [9, 10].

Our data demonstrate a 100% complete success rate of eVAC therapy in the treatment of anastomotic leakages after esophagectomies with gastric pull-up reconstruction. The death of the one patient in the eVAC cohort was not directly related to the anastomotic leakage, as an EGD 1 month prior to his death revealed that the cervical anastomotic leakage the patient was suffering from had healed completely. No patient in the eVAC cohort had to undergo cervical esophagostomy formation. In contrast, ten out of 20 patients (50%) in the non-eVAC cohort had a negative clinical outcome (*p* < 0.05). Of these ten patients, eight underwent cervical esophagostomy formation and three died in the postoperative course due to an insufficiently controlled intrathoracic anastomotic leakage. Our results compare favorably with other international cohort studies [1, 8, 11]. A meta-analysis from 2020 included five retrospective studies with a total of 274 patients treated with either eVAC or SEMS for post-esophagectomy anastomotic leakages [12]. eVAC was associated with a significantly higher rate of leak closure (OR 3.14), a shorter duration of treatment, and a lower mortality rate. Similar results were provided by Rausa and colleagues [13]. This meta-analysis included four studies with a total of 163 patients and also compared eVAC and SEMS treatment for esophageal leaks. The closure rate was significantly higher with eVAC (OR 5.51; *p* < 0.001). Patients treated with eVAC had a shorter treatment duration, lower major complications, and in-hospital mortality compared to SEMS. Furthermore, Mandarino et al. demonstrated a 100% technical success rate of eVAC and a dehiscence closure rate of 75% after failed redo surgery or previous endoscopic treatment in twelve patients with post-esophagectomy anastomotic leakages, which highlights the role of eVAC as a potential salvage therapy [14]. In a recently published study by Chon et al., the safety and feasibility of eVAC in robotic-assisted minimally invasive esophagectomies (RAMIE) was assessed [15]. Twenty-one out of 157 patients developed an anastomotic leakage after Ivor-Lewis RAMIE in the postoperative course. With eVAC as mono-therapy, a closure rate of 75% was achieved. Placement of SEMS after eVAC treatment was performed in four patients due to a persistent leakage. An overall success rate of 95% (19 out of 20 patients) was demonstrated, when different treatment modalities were used, which resembles our findings. Notably, a study by Berlth and colleagues failed to demonstrate superiority of eVAC treatment [16]. In this retrospective study, 76 patients receiving SEMS were compared to 35 patients receiving eVAC after oncologic gastroesophageal surgery. There were no significant differences in overall closure rate (85.7% for eVAC, 72.4% for SEMS; *p* = 0.152), ICU stay, and duration of hospital stay. A possible explanation might be the significant heterogeneity of the patient collective as patients with intraabdominal, intrathoracic, and cervical anastomotic leakages (total gastrectomies ± distal esophagus, Ivor-Lewis esophagectomies, and McKeown esophagectomies) were included in the analysis. However, the heterogeneity of patient collectives is a general problem of all the retrospective studies included in this study. At this point, a prospective study with a randomized, more comparable patient collective is lacking but needed in order to amend this problem.

Furthermore, there is no universal consensus for the treatment of leaking esophagogastric anastomoses. Although eVAC therapy has shown impressive success rates in several studies, the treatment of anastomotic leakages after esophagectomies remains complex and—depending on the size of the leakage and the clinical situation of the patient—may have to be combined with other interventional treatment modalities as a hybrid approach (e.g., fcSEMS, external drainage) or even revision surgery. The elaboration of an individual treatment algorithm requires expert knowledge of surgeons, gastroenterologists, radiologists, and ICU specialists in order to find the best concept in each case. Nonetheless, considering its high efficacy, eVAC therapy should be an integral part of the treatment algorithm, and its application needs be considered in every case of anastomotic leakage as it contributes to the cleanliness of the cavity by removing debris and pus, prevents further leakage, and stimulates the growth of granulation tissue [11, 17, 18]. Even very large defects and defects with a complete dehiscence of the anastomosis were successfully treated with eVAC [19, 20].

In our experience, eVAC therapy is most effective in cases where the sponge is placed through the anastomotic defect right into the cavity behind. In some cases, this may require a gentle enlargement of the existing defect in order to achieve an intracavitary position. An intracavitary placement allows for a better evacuation of debris and pus and even makes an additional/prolonged external drainage unnecessary.

It has been suggested that minor leaks—hence unsuitable for an intracavitary sponge placement—may be treated conservatively with a watch and wait strategy, fibrin glue injection, or clip administration [21–23]. However, these options do not support the cleansing of the perianastomotic tissues. Even in small anastomotic defects, unsuitable for an intracavitary sponge placement, an endoluminal position can be beneficial, although the removal of pus and debris may not be as effective as with an intracavitary placement. Interestingly, it has also been demonstrated that a pre-emptive endoluminal eVAC therapy may reduce the development of anastomotic leakages in the first place [24–26]. In this series, three patients with high-risk anastomoses (macroscopically compromised blood supply of the distal part of the gastric conduit) received prophylactic eVAC therapy and developed an anastomotic leakage in the further course. In all three cases, the integrity of the esophagogastric anastomosis could be maintained/restored through further application of eVAC. In one of these cases, a surgical lavage of the thoracic cavity was performed as part of the treatment concept.

Nevertheless, the entire clinical context needs to be assessed on an individual basis by a multidisciplinary team (MDT) of surgeons, gastroenterologists, radiologists, and ICU specialists. In cases with sufficient external drainage (e.g., via an intraoperatively placed chest tube), primary closure of the defect may be similarly effective and quicker [23]. Usually, the sponge needs to be changed every 3–5 days until the defect has improved sufficiently. The effectiveness of the treatment is assessed at the time of sponge replacement. The eVAC therapy is usually ceased once the defect is clean and the underlying cavity has been downsized enough through the growth of granulation tissue to allow for a safe resumption of oral food intake. In the present study, a median of five cycles of eVAC treatment with a median treatment duration of 18 days was performed. These results are in accordance with the current literature [8, 9]. In case of a clean but persistently large cavity, an additional fcSEMS application may be of use, which was successfully performed in six of our cases.

Moreover, eVAC therapy was associated with a significantly reduced need for surgical revisions in our study (29% vs 65%, *p* < 0.05), which may reflect the efficacy of this treatment approach in controlling and containing sepsis. In the eVAC group, revision surgery was performed solely for drainage and lavage of the thoracic cavity due to abscess formation, pleural empyema, or other fluid collections not amenable for interventional/endoscopic drainage. Pre-existing coronary heart disease was found more often in the eVAC group (6 vs 0 patients, *p* < 0.05). As an underlying cardiovascular disease is generally associated with a high incidence of cardiovascular events in the postoperative course, the lower incidence of death or diversion surgery in this group should be noted [27]. The higher rate of preoperative PPI use in the non-eVAC group may be explained by the higher incidence of GERD and Barrett’s esophagus in this cohort.

It is noteworthy that eight patients in the eVAC cohort had received a cervical anastomosis compared to only one patient in the non-eVAC cohort (*p* < 0.05). Markar et al. demonstrated that the leakage rate in cervical anastomoses is almost five times as high as for intrathoracic anastomoses, which is important considering that the complications of a leakage from a cervical anastomosis may be less severe than from an intrathoracic leakage [28]. However, the rate of treatment failure remains higher in the non-eVAC cohort, even if only the subgroups of patients with an intrathoracic anastomosis are compared (*p* < 0.05).

Notwithstanding the above-mentioned advantages of eVAC treatment, an anastomotic stricture rate of 41% in the eVAC cohort compared to 15% in the non-eVAC cohort could be observed. Although the higher stricture rate in the eVAC cohort did not reach statistical significance, a clear trend was seen. This might be related to excessive granulation tissue stimulated by eVAC treatment and may require repeated dilatations and/or stent application [29]. Yang and colleagues investigated 20 patients, who developed an anastomotic leakage following esophagectomy. eVAC treatment led to a stricture rate of 35%, which is similar to our findings [29].

In the contemporary era, an anastomotic leakage rate of 22% is out of keeping with benchmark data; however, with the evolution of surgical techniques and minimally invasive approaches and in the absence of standardized practices, this will go some way to explain this incidence. Furthermore, since the formalization of operative techniques in the department for the past 5 years, as well as homogeneous approaches to preoperative optimisation, MDT work up, and enhanced recovery after surgery principles, the anastomotic leakage rate has reduced to 6%, which is in line with international benchmark data [1, 2].

A weakness of this study is the heterogeneity of the two study groups with a markedly higher rate of open esophagectomies in the non-eVAC group (95% vs 29%). Several studies comparing open esophagectomies with hybrid approaches found a lower incidence of short-term postoperative complications in the hybrid group, especially in terms of postoperative pulmonary complications [30], while others found no difference or an even higher complication rate with the hybrid approach [31, 32]. To what extent the higher rate of open esophagectomies in the non-eVAC group has contributed to the significantly higher rate of treatment failure in this group is unclear but should be taken into consideration when interpreting the results of this study. The same holds true for the location of the anastomosis with significantly more cervical anastomoses in the eVAC group and the different treatment modalities that were used in both groups. However, prospective, randomized studies are still missing on this topic, and there are only a few retrospective studies comparing eVAC and stent/conventional treatment modalities. Therefore, this study provides further evidence for the significant role of eVAC in esophageal surgery.

## Conclusion

eVAC therapy has shown promise in our series with clear advantages over alternative methods and should be an integral component of the treatment algorithm in these complex scenarios of anastomotic leakages after esophagectomy. A combination of eVAC with other treatment modalities can be beneficial in some cases. Furthermore, each case must be taken on its own individual merits and an MDT approach with adequate clinical, radiological, and surgical expertise to implement the use of these therapies for improving patient outcomes.

## Data Availability

The datasets generated during and/or analysed during the current study are available from the corresponding author on reasonable request.

## Appendix

Table 8.

**Table 8:** Anastomotic leakage

Anastomotic leakage (AL)	eVAC cohort (*n* = 17)	Non-eVAC cohort (*n* = 20)	*P*-value
Indication for the first EGD or CT scan after esophagectomy:			n. s
Elevation of infl. markers	41%	50%	
Clinical deterioration	35%	45%	
Path. secretion of TD	35%	25%	
Prophylactic intention/by chance	18%	0%	
Other	6%*	5%**	

*Air in cervical easy flow drainage

**Preceding bronchoscopy
